# Pedicle screw accuracy in thoracolumbar fractures- is routine postoperative CT scan necessary?

**DOI:** 10.1186/s12891-021-04860-y

**Published:** 2021-11-26

**Authors:** R. Aigner, C. Bichlmaier, L. Oberkircher, T. Knauf, A. König, P. Lechler, S. Ruchholtz, M. Frink

**Affiliations:** 1 Center for Orthopaedics and Trauma Surgery, University Hospital Giessen and Marburg GmbH, Location Marburg, Baldingerstraße, D-35043 Marburg, Germany; 2 Department of Diagnostic & Interventional Radiology, University Hospital Giessen and Marburg GmbH, Location Marburg, Marburg, Germany

**Keywords:** Screw misplacement, Risk factor, Computed tomography, Thoracolumbar fracture

## Abstract

**Background:**

While several studies report on accuracy rates of pedicle screws, risk factors associated with inaccurate pedicle screw positioning in patients with thoracolumbar fractures are reported rarely. CT scan as a routine postoperative control is advocated by various authors, however its necessity remains unclear.

**Methods:**

Two hundred forty-five patients were included in this retrospective study. Percutaneous dorsal instrumentation was most commonly performed (*n* = 201). Classification of Zdichavsky et al. and Rao et al. were used to classify screw misplacement and anterior perforation was further evaluated according to the extent of perforation (< 2 mm; > 2 mm). Multivariate analysis was performed to identify risk factors for misplacement of screws.

**Results:**

One thousand sixty-eight pedicle screws were inserted in 245 patients. Misplacement was found in 51 screws (4.8%) in 42 patients (17.1%) according to the classification of Zdichavsky et al. and in 75 screws (7.0%) in 64 patients (26.1%) according to the classification of Rao et al.. An anterior perforation of the vertebral cortex was found in 56 screws (5.2%). Multivariate analysis showed fracture location in the upper thoracic (*p* = 0.048) and lumbar spine (*p* = 0.013) to be the only independent predictors for screw misplacement. In addition a significant correlation between pedicle diameter and the occurrence of screw malposition was found (*p* = 0.003). No consequences were drawn from postoperative routine CT in asymptomatic patients.

**Conclusion:**

An overall low rate of screw misplacement was found with fracture location in the upper thoracic and lumbar spine being the only factors independently associated with the risk of screw misplacement. No consequences were drawn from postoperative routine CT in asymptomatic patients. Therefore its use has to be discussed critically.

## Background

Dorsal instrumentation is a well-established procedure for stabilization of thoracic and lumbar vertebral fractures and accuracy of pedicle screw placement is required for stable fixation [1, 2]. Open dorsal instrumentation with pedicle screws is still considered to be the gold standard for the treatment of unstable thoracic or lumbar vertebral fractures [3]. Nevertheless, there is an increasing trend towards minimal invasive percutaneous procedures [4, 5].

While there are many studies reporting the rate of accurate placement of pedicle screws [3, 6] studies characterizing the type of misplacement are scarce. Few authors tried to identify factors associated with the risk of screw misplacement. However reports on risk factors for inaccurate screw placement especially in trauma patients are rare in the current literature.

Percutaneous pedicle screw placement is associated with reduced blood loss, less soft tissue damage and shorter operative times [3, 7]. However visualization is limited. Therefore, especially after percutaneous pedicle screw placement, confirmation of accurate screw position with computed tomography or intraoperative 3 D scan seems to be warranted.

Despite the relatively high radiation dosage, computed tomography (CT) scan to confirm accurate screw placement is performed by majority of surgeons [1]. Nevertheless, the question if inaccurate screw positioning in asymptomatic patients is of clinical relevance remains unclear. Therefore the questions of this study are: what are the consequences of postoperative routine CT scan (Question 1)? How is the incidence and type of inaccurate pedicle screw placement (Question 2) and what are risk factors for inaccurate placement of pedicle screws (Question 3)?

## Methods

All consecutive patients ≥18 years diagnosed with and treated for thoracic or lumbar vertebral fracture between January 2011 and 2017 were retrospectively identified and screened for inclusion criteria.

The diagnosis of the vertebral fracture was based on plain radiographs and a thin- slice CT was performed in all patients for exact understanding of the fracture pattern as well as pre- operative planning (pedicle diameter and screw lengths). Patients were excluded by the following exclusion criteria: 1. age < 18 years; 2. initial treatment in another hospital; 3. missing postoperative CT scan; 4. degenerative indications without acute trauma. Fractures were classified according to the AOSpine thoracolumbar spine injury classification system [8] and demographic data including age, gender, body mass index (BMI), comorbidities assessed using the Charlson Comorbidity index [9], ASA Score [10] and Injury Severity Score (ISS) [11] were recorded. Complications were recorded and classified according to the classification of Dindo et al. [12].

The standard surgical procedure was percutaneous fluoroscopy aided dorsal instrumentation. Surgery was performed under general anesthesia with the patient placed in prone position on a carbon radiolucent operating table. One image intensifier was used for fluoroscopy guided pedicle screw placement. Skin incision was placed 1–2 cm lateral to the pedicle allowing angulation considering the anatomy of the pedicle known from the pre- operative performed CT. Osseous insertion of the Jamshidi needle was performed on the lateral margin of the pedicle in the a.p. projection. When the Jamshidi needle reached the posterior wall of the vertebral body in the lateral projection it was aimed to be lateral to the medial wall of the pedicle in the a.p. projection. A guidewire was then placed through the cannulated Jamshidi needle and the cortical structure of the pedicle was drilled after dilatation of the soft tissue. Lengths and diameter of the screws used were determined according to preoperative CT scans. Only polyaxial screws were used. Longitude and Sextant systems (Medtronic, Minneapolis, USA) were used for dorsal instrumentation. Patients with spinal canal stenosis > 30% and/ or neurological symptoms were treated with open laminectomy following open dorsal instrumentation. The patients included in the present study were treated by a total of eleven different surgeons. As a standard, these surgeries were performed only in the presence of a senior physician in our department. Patients with preoperative neurologic symptoms were treated according to the NASCIS-II scheme [13]. Antibiotic prophylaxis was performed with second generation cephalosporin. All patients were mobilized with full weight bearing beginning from day one after surgery. Venous thromboembolism prophylaxis was performed with low molecular weight heparin until full mobilization was achieved.

**Fig. 1:**
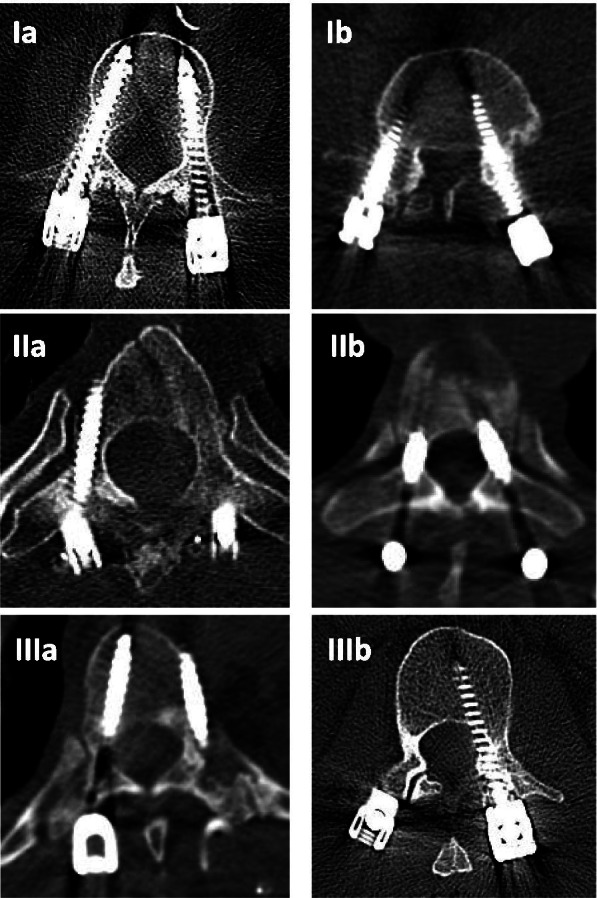
Exemplary CT scans showing the different grades of Zdichavsky classification

As a standard procedure postoperative CT scan was performed in patients treated for thoracic or lumbar vertebral fractures. Evaluation of screw accuracy was performed using two different grading systems. Firstly, classification of Zdichavsky et al. was used [14] (see Fig. 1). Furthermore, the classification proposed by Rao et al. [15] was used. This classification rates screw misplacement according to the perforation of the pedicle (Grade 0: no perforation of the pedicle; 1: less than 2 mm; 2: 2 to 4 mm; 3: greater than 4 mm). Additionally anterior perforation of the vertebral body was classified as A: no anterior perforation; B: anterior perforation < 2 mm; C: > 2 mm anterior perforation.

Data were collected, and the dataset was deidentified using an Excel 2007 database (Microsoft Corp, Redmond, WA, USA). For statistical analysis, SPSS Statistics 22 (IBM Corp, Armonk, NY, USA) was used for the explorative data analyses, descriptive statistics were used to describe the clinical characteristics and misplacement rates. Data are presented as the mean ± standard deviation. All items were included in a multivariate binary logistic regression analysis to identify independent risk factors on a patient level (risk factors for a patient to have a screw misplaced). Misplacement according to the classification of Zdichavsky et al. was used for statistical analysis. A *P* value of <.05 was considered to be significant.

## Results

### Demographic parameters

**Fig. 2:**
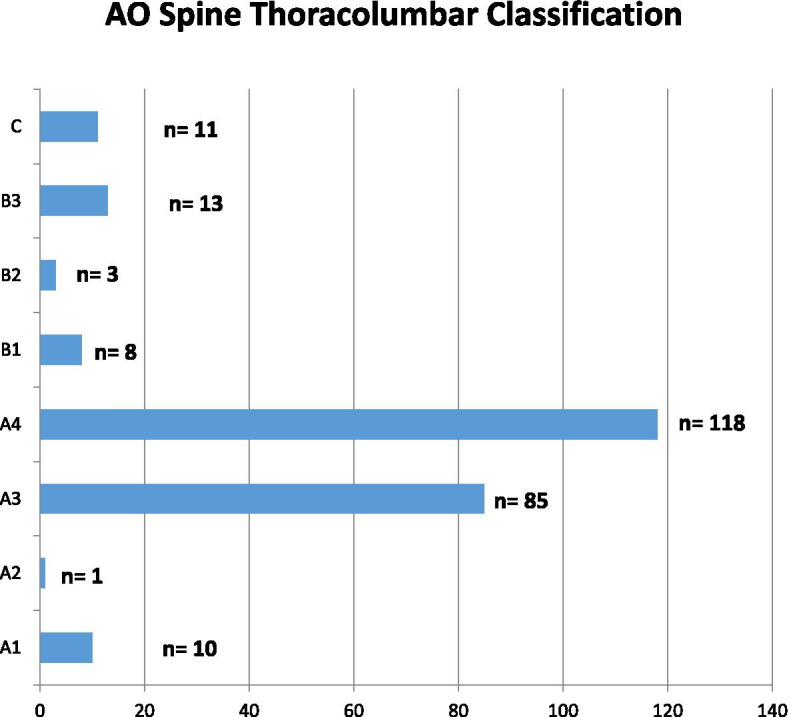
Fracture classification according to AO Spine Thoracolumbar Classification

Overall, 245 patients met the inclusion criteria and were included. Demographic parameters are shown in Table 1, fracture classification is depicted in Fig. 2. Two hundred one patients (82.0%) were treated with percutaneous dorsal instrumentation and the remaining 44 patients (18.0%) had open laminectomy and dorsal instrumentation. Surgeries of 193 patients (78.8%) were scheduled during regular operating hours and 52 patients (21.2%) had emergency surgeries on nightshifts and weekends. A total of 28 patients (11.4%) received additional stabilization of the anterior column. This was conducted in a one staged procedure in two patients and in a two staged procedure (mean time period between first and second procedure 8.2 ± 12.4 days) in 26 patients. Screw cement augmentation was performed in 69 patients (28.2%).

**Table 1:** Demographic parameters

Age	57.5 ± 19.8 years
Gender (male/ female)	95/150
BMI	26.4 ± 5.0 kg/m^2^
Charlson Score	1.4 ± 2.1
ASA Score	2.3 ± 0.8
ISS	15.2 ± 10.8
ISS > 15	*N* = 76 (31%)
Reason for dorsal Instrumentation	
Traumatic vertebral fracture	*N* = 224 (249 fractured vertebral bodies)
Pathological fracture	*N* = 16 (19 fractured vertebral bodies)
Spondylodyszitis	*N* = 5
Fracture location	
upper thoracic spine (Th1-Th5)	*N* = 17 (6.9%)
lower thoracic spine (Th6-Th10)	*N* = 39 (15,9%)
thoracolumbar junction (Th11–L2)	*N* = 150 (61.2%)
lumbar spine (L3-L5)	*N* = 39 (15.9%)

Overall, 53 patients (21.6%) suffered a total of 62 complications. Most of the complications (*n* = 32) were grade II complications (nosocomial infections, uncomplicated renal failure and others). In total, 19 grade III complications were observed (screw misplacement, hematoma, pleural effusion and others). Seven grade IV complications including myocardial infarction, pulmonary embolism and others and four grade V complications were recorded. The causes of death during hospitalization were septic multiorgan failure after nosocomial infection in two cases, cardiovascular failure in a polytrauma patient with preexisting congestive heart disease in one case, and cardiovascular failure after myocardial infarction in one case.

Surgery-associated revision procedures were necessary in a total of 7 patients (2.9%). These were four revisions due to screw malposition, two due to clinical relevant hematoma, and one due to wound infection.

### Evaluation of the consequences of postoperative routine CT scan (question 1)

In four patients (1.6%), operative revision procedures were performed after identification of inaccurate screw positioning in postoperative computed tomography scans. Three of them (all classified as grade IIIb according to Zdichavsky et al.) showed new neurological symptoms after surgical treatment all classified as grade D according to The American Spinal Injury Association (ASIA) impairment scale, while one asymptomatic patient was revised during a planned pelvic surgery 3 days after dorsal instrumentation to improve screw positioning. In all three patients, neurologic deficits improved after screw correction but persisted until discharge.

No consequences from identification of anterior perforation on postoperative CT were detected. Vascular or visceral complications associated with an anterior perforation of the vertebral body did not occur in the analyzed population.

### Incidence and type of inaccurate pedicle screw placement (question 2)

One thousand sixty-eight pedicle screws were inserted in 245 patients. One thousand seventeen screws (95.2%) were classified as Ia according to the classification of Zdichavsky et al.. Misplacement was found in 51 screws (4.8%) according to the classification of Zdichavsky et al. with 19 screws classified as Ib, two screws as IIa, 14 screws as IIb, three screws as IIIa and 13 as IIIb. Overall screw misplacement occurred in 42 patients (17.1%).

According to the classification of Rao et al., 75 screws (7.0%) perforated the pedicle wall. Forty-six screws were minor perforations (< 2 mm) classified as grade 1, 15 were classified as grade 2 (2–4 mm) and 14 were classified as grade 3 (> 4 mm). Forty-eight screws breached the medial and 27 the lateral cortex of the pedicle. Following this classification 64 patients (26.1%) showed inaccurate screw positions.

An anterior perforation of the vertebral cortex was found in 56 screws (5.2%). Fifty screws perforated the anterior cortex less than 2 mm while 6 screws showed an anterior perforation > 2 mm. The six screws that showed anterior perforation > 2 mm were located in the 5th and 7th thoracic vertebrae in one case each, and in the 3rd lumbar vertebra and the 1st sacral vertebra in two cases each.

### Risk factors for inaccurate placement of pedicle screws (question 3)

No differences were found in the rate of screw misplacement between men and women (*p* = 0.345). Mean age did not significantly differ between patients with and without inaccurate screws (*p* = 0.381). Significant differences in pedicle screw accuracy rates were found with regard to the location of the fracture (*p* = 0.015). BMI did not differ between the groups with and without inaccurate placed screws (*p* = 0.969). On average, in patients who had at least one screw inaccurately placed overall more screws were implanted (*p* = 0.005). After open pedicle screw insertion misplacement was significantly more common compared to percutaneous treatment (*p* = 0.049).

Multivariate analysis showed fracture location in the upper thoracic (Th1-Th5) (*p* = 0.048) and lumbar spine (*p* = 0.013) to be the only independent predictors for screw misplacement after adjustment for the other potential risk factors (see Table 2).

**Table 2:** Association between considered variables and screw misplacement according to the classification of Zdichavsky et al

Covariate	OR(95%CI)	*p*-value	Global *p*-value
Number of screws	1.21 (0.87,1.67)		0.255
**Fracture localization**			**0.038**
Thoracolumbar junction	reference		
**Upper Thoracic spine**	**3.93 (1.01,15.27)**	**0.048**	
Lower Thoracic spine	2.21 (0.77, 6.37)	0.142	
** Lumbar spine**	**3.41 (1.30,8.95)**	**0.013**	
Open/Percutaneous			0.618
Open	reference		
Percutaneous	0.78 (0.29,2.10)		
Gender			0.222
male	reference		
female	1.62 (0.75,3.51)		
Age	1.01 (0.99,1.03)		0.513
BMI	1.03 (0.96,1.10)		0.488

Analysis at the screw level showed a significant correlation between pedicle diameter and the occurrence of screw malposition (*p* = 0.003). Inaccurately placed screws had a significantly smaller mean pedicle diameter than correctly placed screws (7.47 ± 3.17; range 2–17 mm vs. 7.97 ± 2.10; range 3–18 mm; p = 0.003). Furthermore, there was a significant difference in the number of screw failures depending on the height at which the screw was inserted (T1-T5: 11.11%; T6-T10: 6.93%; T11-L2: 2.72%; L3-S1: 5.36%; *p* = 0.002).

## Discussion

The results of the current study showed a low overall rate of inaccurate placed screws in patients mainly treated with percutaneous dorsal instrumentation and fracture location was the only independent factor associated with screw misplacement. No consequences were drawn from postoperative routine computed tomography in asymptomatic patients except in one specific case.

### Evaluation of the consequences of postoperative routine CT scan (question 1)

In this sample, a total of 13 screws were classified as IIIb and further 14 screws as IIb according to the classification of Zdichavsky et al. [14]. However, revision surgery due to malpositioned screws was performed in only four patients, showing new onset neurological symptoms after dorsal instrumentation in three patients. These numbers confirm that screw misplacement even involving the spinal canal is not necessarily associated with neurological symptoms. However, it must be critically discussed that neurological symptoms may also develop at a later time point, which could not be assessed in this study due to the study design. Therefore some authors recommend removal of any pedicle screw misplaced totally within the spinal canal regardless of the severity of spinal canal intrusion [16]. No further consequences were drawn from postoperative CT in this sample. Considering the significant radiation dosage and additional costs caused by computed tomography its routine postoperative use after dorsal instrumentation has to be discussed critically. Especially in light of the increasing use of intraoperative 3 D scans and navigated placement of pedicle screws, the need for postoperative CT diagnostics to control screw position must be critically reconsidered in the future.

### Incidence and type of inaccurate pedicle screw placement (question 2)

This study revealed an overall accuracy rate of 95.2% of mainly percutaneously inserted pedicle screws according to the classification of Zdichavsky et al. and 17.1% of the patients included had at least one screw misplaced. These numbers are in line with the current literature. Tinelli et al. found an overall misplacement rate of 6.3% of the screws in 17.3% of the patients [3]. Raley and Mobbs found 9.7% of percutaneously inserted pedicle screws to be misplaced. However, comparison to the results of the current study is limited by the fact that patient populations in these studies mostly included degenerative cases and pedicle perforation was assessed only using a grading system similar to the classification proposed by Rao et al. [17].

While accuracy rates have been reported frequently in the current literature the type of misplacement is rarely reported. In line with the results of the present study, medial pedicle wall perforation was more common in a study of Mohanty et al. [18]. Zdichavsky et al. showed grade III to be the most common type of misplacement (8.6%) with an almost equal ratio of IIIa and IIIb classified screws and an overall misplacement rate including also grade Ib screws of 18.4%, analyzing 278 pedicle screws in patients with thoracic vertebral fractures [19]. In contrast, in the present study, pedicle screw placement lateral outside the pedicle with the tip of the screw inside the vertebral body (grade Ib) was more common and more grade IIIb than IIIa classified screws were detected indicating that serious pedicle violation more frequently occurred medially. This could be caused by the fact that Zdichavsky et al. only included thoracic fractures. Furthermore, mostly percutaneous dorsal instrumentation was performed in our sample whereas open dorsal instrumentation was performed in the study by Zdichavsky et al.. The assumption that medial pedicle perforation is more common in percutaneous pedicle screw placement is supported by the study of Oh et al. [20]. In this retrospective case series of 1056 pedicle screws, the incidence of medial penetration was significantly increased in the percutaneous group, while lateral penetration was more common in the open group [20].

Malpositioning of pedicle screws can not only cause nerve root or spinal cord injury in case of pedicle wall breach, but also visceral or vascular injury in case of anterior perforation. In this study 5.2% of the screws showed an anterior perforation although no associated complications were observed. This is in line with the current literature examining the accuracy of percutaneous pedicle screws. Heintel et al. showed a rate of 4.8% of pedicle screws with anterior perforation without complications or required surgical revision [6]. Foxx et al. showed that 33 of 680 pedicle screws were in contact with a major vessel proven in postoperative imaging. After a mean follow up of 44 months, these patients did not develop any complications associated with vascular injuries [21]. However potential consequences such as aortic perforation have to be considered since they have been described in the literature [22, 23].

### Risk factors for inaccurate placement of pedicle screws (question 3)

Few authors tried to determine risk factors for screw misplacement [24, 25]. In line with a previous report [25], age, sex and BMI were not associated with the rate of misplaced screws in the presents study. However, regarding the fracture location significant differences were observed in our sample between the upper thoracic, lower thoracic, thoracolumbar and lumbar spine showing less misplaced screws in the thoracolumbar junction and the lower thoracic spine. Jin et al. identified the middle thoracic spine (Th5–8) to be at increased risk for screw misplacement in scoliosis surgery [24]. The fact that pedicle screw misplacement occurred more frequently in the upper thoracic and lumbar spine could be explained by decreased pedicle diameters and subsequent technical difficulty. Furthermore, experience of the surgeon could be reasonable as in our sample in line with previous studies [6] most of the fractures were located in the thoracolumbar junction (Th11–L2) representing the typical area of vertebral fractures. In accordance with the current literature, this study did not identify differences regarding the pedicle screw accuracy between open and percutaneous dorsal instrumentation in the multivariate analysis [20, 26].

### Strengths and limitations

The results of the present study are limited by several factors. The most important limitation is the retrospective study design. Although patients’ records were carefully reviewed, data are strongly dependent on the quality of documentation. In addition, some parameters, such as the presence of osteoporosis, cannot be collected in a retrospective study. Moreover a limitation of this study is that pain situation could not be assessed due to the retrospective study design. Furthermore, we did not provide long-term follow-up data and therefore we cannot exclude symptoms of instability or vascular complications after hospitalization in patients with misplaced screws. Finally, because of the exclusion criteria mentioned above, no statements can be made about patients < 18 years of age and about patients with degenerative diagnoses.

Strength of the present study is the high number of patients from a high-volume center with standardized treatment algorithm. A multivariate analysis was performed to identify independent risk factors for screw misplacement.

## Conclusion

This study showed an overall low rate of screw misplacement in mainly percutaneously treated patients with thoracic and lumbar vertebral fractures. Fracture location showed to be the main factor associated with screw misplacement with upper thoracic and lumbar fracture location being at increased risk. In addition a significant correlation between pedicle diameter and the occurrence of screw malposition was found. No consequences were drawn from postoperative routine CT scans to control for accurate pedicle screw placement in asymptomatic patients. Therefore, we believe that the use of a postoperative CT scan should be critically discussed and should be reserved for symptomatic patients.

## Data Availability

The datasets generated and analyzed during the current study are not publicly available due to limitations of ethical approval involving the patient data and anonymity but are available from the corresponding author on reasonable request.
